# Variation in Diffusion Tensor Imaging Parameters in the Cervical and Thoracic Spinal Cord (C1-C5 and C6-T2) Segments of Normal Beagle Dogs

**DOI:** 10.3390/vetsci10010031

**Published:** 2023-01-01

**Authors:** Kiyotaka Arai, Takamasa Itoi, Natsuki Akashi, Masahiro Miyabe, Keisuke Sugimoto, Akira Matsuda, Noritaka Maeta, Teppei Kanda, Kenji Kutara

**Affiliations:** Faculty of Veterinary Medicine, Okayama University of Science, Ikoinooka 1-3, Imabari 794-8555, Ehime, Japan

**Keywords:** diffusion tensor imaging, spinal cord, dog, fractional anisotropy

## Abstract

**Simple Summary:**

Diffusion tensor imaging (DTI) is a specialized imaging technique that measures the strength and vector of water molecule movement, which is analyzed using a variety of parameters. This method has the potential to depict pathological conditions that cannot be studied using conventional magnetic resonance imaging methods. However, most reports on DTI parameters measured only fractional anisotropy and apparent diffusion coefficient values in veterinary medicine. Therefore, we performed DTI on five adult beagles under anesthetic maintenance and analyzed the various DTI parameters obtained. After post-processing, DTI parameters were calculated along the entire spinal cord. Among DTI parameters, fractional anisotropy, relative anisotropy, and axonal diffusivity significantly decreased in the caudal direction. However, the apparent diffusion coefficient, radial diffusivity, and mean diffusivity values were not significantly correlated with vertebral levels. We provide evidence on the existence of segment-dependent DTI parameters in the canine cervical spinal cord. Therefore, comparisons of DTI parameters between lesions at different vertebral levels should be avoided unless normative data are available. Furthermore, the DTI data obtained in this study may contribute to the development of a clinical reference for spinal cord evaluation in dogs using DTI parameters.

**Abstract:**

This study aimed to determine the characteristics and reference values of each vertebra in the cervicothoracic region by performing diffusion tensor imaging (DTI) scans and analyzing DTI parameters in normal Beagle dogs. In five adult Beagles under anesthetic maintenance, DTI was performed using a 1.5-T magnetic resonance imaging (MRI) scanner. Axial DTI was performed using three overlapping slabs to cover the cervical and thoracic spinal cords. After post-processing, DTI parameters were calculated along the entire spinal cord. Among DTI parameters, fractional anisotropy, relative anisotropy, and axonal diffusivity significantly decreased in the caudal direction. However, the apparent diffusion coefficient, radial diffusivity, and mean diffusivity values were not significantly correlated with vertebral levels. We provide evidence for the existence of segment-dependent DTI parameters in the canine cervical spinal cord. Therefore, comparisons of DTI parameters between lesions at different vertebral levels should be avoided unless normative data are available. Furthermore, the DTI data obtained in this study may contribute to the development of a clinical reference for spinal cord evaluation in dogs using DTI parameters.

## 1. Introduction

Diffusion tensor imaging (DTI) is a special imaging technique that measures the strength and vector of movement of water molecules. It has the potential to observe pathological conditions that cannot be investigated with conventional magnetic resonance imaging (MRI) methods. Normally, water molecules undergo random Brownian motion, known as isotropic diffusion. However, in vivo, especially in axonal fibers, water molecules demonstrate enhanced diffusion in a certain direction along the fibers. This is anisotropic diffusion. Because axon fibers generally exhibit stronger anisotropic diffusion when in the direction of the fiber than when perpendicular to the fiber, it is possible to measure not only the direction of the axon fiber but also the degree of myelination and demyelination due to increased or decreased anisotropy. In DTI, which measures the anisotropic diffusion coefficient, the commonly used measures are fractional anisotropy (FA), which describes the degree of directional dependence; mean diffusivity (MD) and apparent diffusion coefficient (ADC), which express the magnitude of diffusion; and relative anisotropy (RA), which describes the ratio of the anisotropic part to its isotropic part. DTI can also be used to measure parameters such as axonal diffusivity (AD), which represents the diffusivity of water parallel to the axon fiber, and radial diffusivity (RD), which represents the diffusivity of water perpendicular to the axon fiber. These parameters have been extensively studied in humans, including standardization and variation at the vertebral level [1,2,3,4,5,6].

DTI has also been investigated in veterinary medicine for its usefulness in various neurological diseases [7,8,9,10]. However, most reports on DTI parameters measured only FA and ADC values [7,10]. To our knowledge, few reports have examined in detail the variation in DTI parameters at each vertebral level in the thoracolumbar spine and clinical cases of degenerative myelopathy [8]. The cervicothoracic region–C1-T2 segments–is a common site of neurological diseases, similar to the thoracolumbar region–the T3-L3 and L4-S1 segments [11]. Using DTI data of the cervicothoracic region, several reports have compared diseased spinal cords with normal cords [7,10]. However, to our knowledge, no study has compared the normal DTI parameters of the spinal cord at each vertebral level in the cervicothoracic region.

This study aimed to determine the characteristics and reference values of the spinal cord at each vertebra in the cervicothoracic region by performing DTI scans and analyzing DTI parameters in normal Beagle dogs.

## 2. Materials and Methods

### 2.1. Study Design and Animals

This prospective study strictly followed the animal care rules of the Laboratory Animal Center, Imabari Campus, Okayama University of Science, and was compliant with the Guide for the Care and Use of Laboratory Animals (8th ed.). The experimental protocols were approved by the Animal Care and Use Committee of the Okayama University of Science (approval number: 2021-037).

Five adult Beagle dogs (two males and three females, 8.2−11 kg, 2 years old) free of neurological symptoms were enrolled in this study. These animals had not been included in any previous research. The dogs were housed in the Laboratory Animal Center at the Imabari Campus, Okayama University of Science, under a 12:12-h light-dark cycle (light period, 8:00 a.m. to 8:00 p.m.). Room temperature was maintained between 24 °C and 26 °C, and humidity was between 40–60%. For inclusion, all dogs were clinically healthy based on physical examination and complete blood count, and results from biochemical testing, abdominal radiography, and echocardiography were normal. The animals were fasted for approximately 12 h prior to anesthesia. For the anesthetic protocol, propofol at a dose of 6–7 mg/kg was administered intravenously (IV), and the dogs were intubated using a tracheal tube. General anesthesia was maintained using sevoflurane (2.5%; vaporizer setting) and oxygen (2 L/min) under mechanical ventilation (respiratory rate, 10 breaths/min; respiratory pressure, 10–15 cm H_2_O; and end-expiratory carbon dioxide concentration, 40 mmHg). Needle catheters (22 G) were placed in the right or left cephalic veins to administer a propofol injection.

### 2.2. MR Imaging Protocol

MRI scans were performed using a 1.5-T MR imaging scanner (Vantage Elan, Canon Medical Systems, Otawara, Japan) with a 4-channel knee matrix and 4-channel spine matrix coil. The protocol comprised an initial T2-weighted image of the sagittal plane of the cervical and thoracic cords. Transverse DTI scans were obtained.

**Figure 1:**
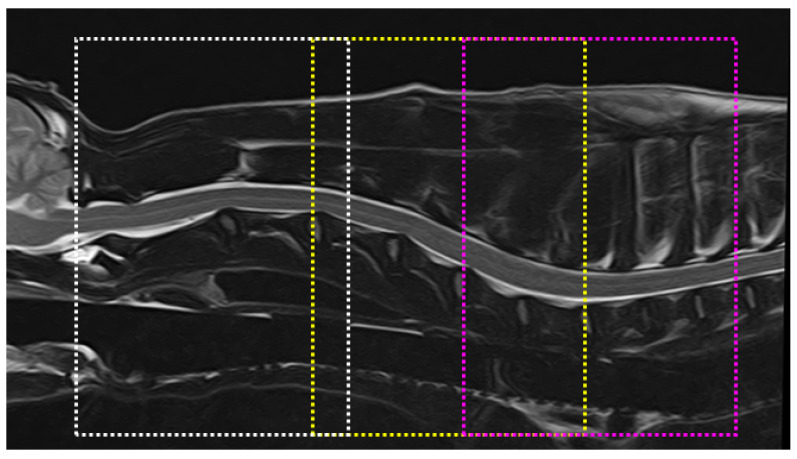
Transverse diffusion tensor images acquired using three overlapping slabs in T2-weighted images in the sagittal plane to cover the C1-C4 (white line box), C4-C7 (yellow line box), and C6-T2 vertebrae (pink line box).

Transverse diffusion tensor images were acquired using three overlapping slabs in the same anatomic location prescribed for T2WI to cover the C1-C4, C4-C7, and C6-T2 segments (Figure 1). The imaging parameters for each slab of DTI acquisition included the following: field of view, 22 × 22.5 cm; matrix, 160 × 160; voxel size, 1.37 × 1.4 mm^3^; slice thickness, 3 mm; repetition time, 5000 ms; and *b*-value, 1000. Diffusion-weighting gradient schemes with 12 non-collinear directions were used.

Total MRI scanning time was approximately 1 h.

### 2.3. Image Analysis

**Figure 2:**
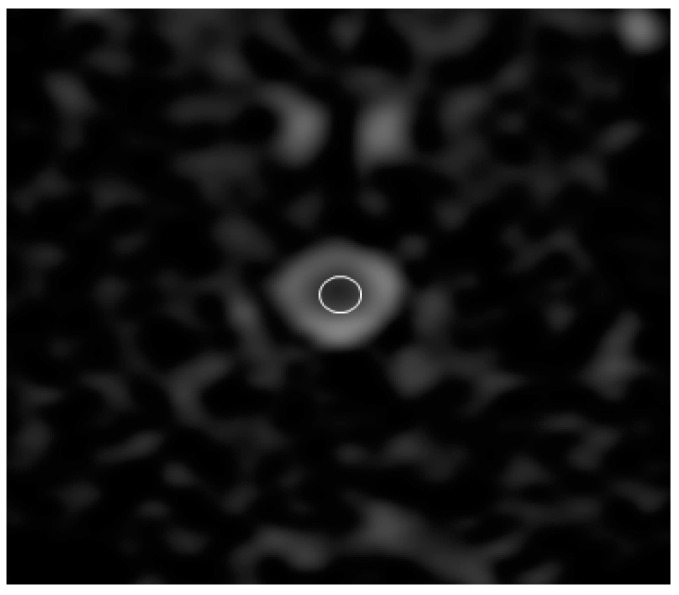
Apparent diffusion coefficient image of the C5 vertebra. The region of interest (ROI) is placed manually on the spinal cord with special care to include the least amount of cerebrospinal fluid. ROI size was standardized to 0.3 cm^2^.

Sagittal T2 images were used to identify the relevant anatomy. ADC, FA, RA, and three types of diffusion eigenvalue (λ1, λ2, and λ3) maps were created using the package built on the MRI system. To measure the ADC, FA, RA, λ1, λ2, and λ3 values, the regions of interest (ROIs) were placed manually on the spinal cord in all transverse images with special care to include the least amount of cerebrospinal fluid (Figure 2). The size of the ROI was standardized to 0.3 cm^2^. For ADC, FA, RA, λ1, λ2, and λ3 values, these data were analyzed using a workstation built into the MRI system. The λ1, λ2, and λ3 values were used to calculate DTI parameters using the following formula:

AD = λ1(1)

RD = (λ2 + λ3)/2(2)

MD = (λ1 + λ2 + λ3)/3(3)

Each DTI value was averaged for each vertebral level.

### 2.4. Statistical Analysis

All DTI values are represented as mean ± standard deviation. Statistical tests were performed using commercially available statistical analysis software (Stat Mate III; ATMS Co., Tokyo, Japan). The Spearman’s rank test was used to analyze the relationships between DTI values and body weight. Additionally, the correlation between the DTI values for the spinal cord at each vertebra was evaluated using the Spearman’s rank correlation coefficient, and the correlation coefficient (r_s_) was calculated. A *p*-value < 0.05 was considered significant. A correlation coefficient >0.5 was defined as a positive correlation, whereas that <−0.05 was defined as a negative correlation.

## 3. Results

All DTI data are summarized in Table 1. None of the DTI data showed a significant correlation with body weight. FA, RA, and AD values were decreased in the caudal direction and were found to be significantly negatively correlated with vertebral level (FA, *p* < 0.001 and rs = −0.75; RA, *p* < 0.001 and rs = −0.79; and AD, *p* < 0.001 and rs = −0.47). ADC, RD, and MD values were not significantly correlated with vertebral levels.

**Figure 3:**
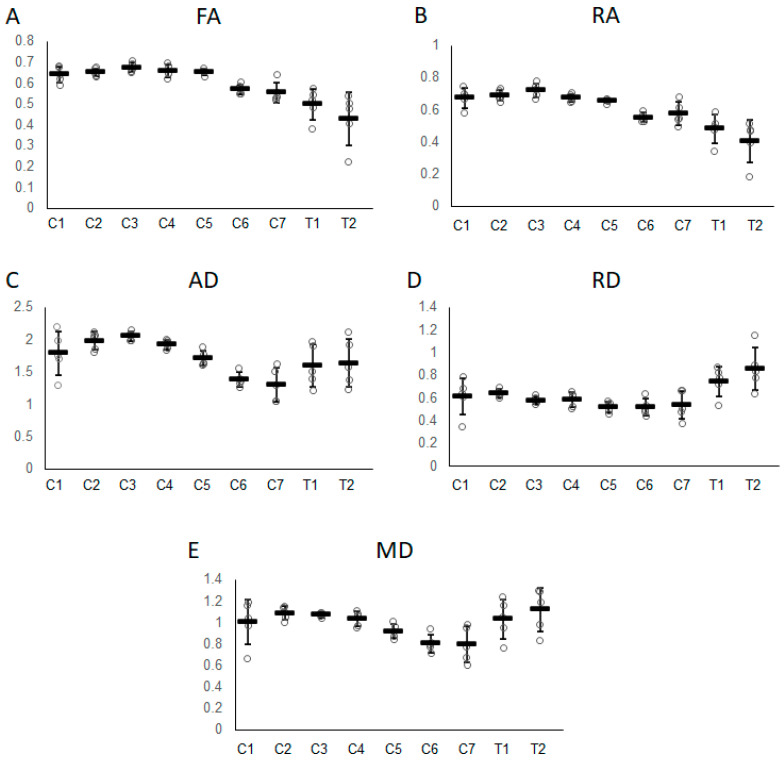
Graphs representing variations in (**A**) FA, (**B**) RA, (**C**) AD, (**D**) RD, and (**E**) MD values from the C1 to T2 vertebral levels. Error bars represent standard deviations.

The maximum and minimum FA values in the DTI parameters for each vertebra are indicated by the C3 and T2 segments, respectively (Figure 3A). The maximum values of RA and AD are indicated by C3 (Figure 3B,C). The minimum values of RA were indicated by T2 (Figure 3B), whereas that of AD was indicated by C7 (Figure 3C). In contrast to FA and RA, AD slightly increased when moving from segments C7 to T2. RD was indicated as the minimum value by C6 and gradually increased toward the maximum value at T2 (Figure 3D). MD gradually decreased from C2 and showed a minimum value at C7. MD suddenly increased from C7 and peaked at T2 (Figure 3E). The ADC values obtained from the ADC map were almost identical to the MD values at all vertebral levels.

## 4. Discussion

This study demonstrated that DTI parameters are inconsistent throughout the cervicothoracic cord. Among DTI parameters, FA, RA, and AD values significantly decreased in the caudal direction. Generally, in cervical segments, the ratio of white-to-gray matter is the highest cranially and then gradually decreases as it progresses caudally along the spinal cord [12]. In a previous study on dogs, FA, RA, and AD values were significantly higher in the white matter than in the grey matter [13]. Accordingly, the results of this study suggest a decrease in the white-to-gray matter ratio as it progresses caudally along the spinal cord.

To our knowledge, this study is the first to evaluate canine DTI parameters at the cervical and upper thoracic vertebral levels as previous studies have been conducted on humans and rodents. Consistent with the findings of this study, a decreasing trend in FA, RA, and AD values toward the posterior part of the cervical spinal cord has been reported in some human studies [2,3,4,5,6]. However, in humans, AD has been reported to consistently decrease from the cervical to the upper thoracic spinal cord [5]. This result is different from that reported in the dogs in this study where AD showed a decreasing trend toward the posterior cervical spinal cord and an increasing trend toward the upper thoracic spinal cord. Furthermore, FA values in the spinal cord of mice and rats are almost the same in the cervical and thoracic spinal cord, which differs from that in dogs and humans [14,15]. These findings suggest that normal variation in DTI parameters differs in varying degrees among species due to anatomical and histological differences.

In addition to segment-dependent variation, age has been reported to influence DTI parameters in humans. Fiber number, axonal diameter, and fiber density decrease with age [16], resulting in a decrease in the FA value [4]. In contrast, gender differences in microstructure have rarely been reported in humans. Thus, it has been suggested that DTI parameters do not differ between genders [4]. In this study, no differences in DTI parameters between genders were observed, but we did not study age-related variations. A study with a larger sample size is needed to clarify these factors in dogs.

In this study, cardiac triggering/gating was not employed because of the significant increase in scan time. This decision was based on previous reports that cardiac triggering/gating does not improve DTI quality in the human spinal cord [3,6]. However, the closer to the thoracic cavity, the larger the standard deviation of each DTI parameter value, suggesting that cardiac motion may have an effect. The cervical and upper thoracic spinal cord were found to have vibrated owing to heartbeat during scanning in humans [17]. Furthermore, it has been reported that image acquisition during the quiescent phase of cardiac-related cord motion resulted in greater consistency in the dominant direction of the diffusion tensor laying and reduced artifacts [18]. Thus, if a shorter scanning time can be expected (e.g., by installing high-performance equipment), it may be useful to use cardiac triggering or gating to avoid value variability.

This study has a limitation: the whole spinal cord was evaluated without distinction between white and gray matter. It was difficult to identify the spinal cord structures from the blurred images obtained using a 1.5-T MRI. In a human study using a 3-T MRI, it has been reported that, in the normal cervical spinal cord, FA values in the white matter, but not gray matter, were reduced caudally [19]. Fiber diameter and volume increase toward the lower vertebra level within the human cervical spinal cord [16]. Moreover, myelinated axons decrease from the middle cervical spinal cord toward the caudal direction [20]. These microstructural differences are thought to result in differences in fiber density and water diffusivity, leading to variations in DTI parameters throughout the human white matter and spinal cord. It is unknown whether similar microstructural changes occur in the canine spinal cord that affects DTI parameters. Thus, histological analysis or ultra-high magnetic field MRI is needed to clarify the causes of DTI parameter variation in dogs.

In summary, an initial understanding of dog DTI parameters was described in normal cervical and upper thoracic spinal cords, which showed a clear trend of decreased FA, RA, and AD in the caudal direction. Because DTI parameters in the canine spinal cord exhibit segment-dependent variability, comparisons of DTI parameters between lesions at different vertebral levels should be measured with this considered. We believe our results will contribute to the development of a clinical reference for canine spinal cord evaluation using DTI parameters.

## Figures and Tables

**Table 1:** Diffusion tensor imaging parameters at each vertebral level.

	C1	C2	C3	C4	C5	C6	C7	T1	T2
ADC (×10^−3^ mm^2^/s)	1.01 ± 0.20	1.10 ± 0.06	1.07 ± 0.02	1.02 ± 0.08	0.91 ± 0.08	0.80 ± 0.12	0.75 ± 0.17	1.00 ± 0.18	1.10 ± 0.21
FA	0.64 ± 0.04	0.66 ± 0.02	0.68 ± 0.03	0.66 ± 0.03	0.65 ± 0.05	0.58 ± 0.02	0.55 ± 0.05	0.49 ± 0.07	0.41 ± 0.13
RA	0.67 ± 0.06	0.70 ± 0.03	0.73 ± 0.05	0.68 ± 0.02	0.66 ± 0.06	0.56 ± 0.03	0.58 ± 0.07	0.47 ± 0.09	0.39 ± 0.13
AD (×10^−3^ mm^2^/s)	1.81 ± 0.34	2.01 ± 0.12	2.07 ± 0.08	1.92 ± 0.10	1.69 ± 0.19	1.38 ± 0.16	1.22 ± 0.26	1.52 ± 0.33	1.57 ± 0.37
RD (×10^−3^ mm^2^/s)	0.61 ± 0.16	0.64 ± 0.05	0.58 ± 0.04	0.58 ± 0.07	0.51 ± 0.04	0.51 ± 0.10	0.51 ± 0.13	0.74 ± 0.13	0.87 ± 0.19
MD (×10^−3^ mm^2^/s)	1.01 ± 0.21	1.10 ± 0.06	1.08 ± 0.02	1.03 ± 0.07	0.91 ± 0.08	0.80 ± 0.12	0.75 ± 0.17	1.00 ± 0.18	1.10 ± 0.21

The data shown are mean value ± standard deviation. AD, axonal diffusivity; ADC, apparent diffusion coefficient; FA, fractional anisotropy; MD, mean diffusivity; RA, relative anisotropy; RD, radial diffusivity.

## Data Availability

Not applicable.
